# Follow-up of incidentally detected mild to moderate ascending aortic dilation and risk factors for rapid progression in a Swedish middle-aged population

**DOI:** 10.1136/heartjnl-2024-325409

**Published:** 2025-03-13

**Authors:** David Kylhammar, Fredrik Nilsson, Petter Dyverfeldt, Filip Hammaréus, Lena Jonasson, Aleksandra Trzebiatowska-Krzynska, Marcus Lindenberger, Lennart Nilsson, Fredrik Nyström, Chiara Trenti, Jan Engvall, Eva Swahn

**Affiliations:** 1 Department of Clinical Physiology, and Department of Health, Medicine and Caring Sciences, Linköping University, Linkoping, Sweden; 2 Wallenberg Centre for Molecular Medicine, Linköping University, Linkoping, Sweden; 3 Department of Cardiology, and Department of Health, Medicine and Caring Sciences, Linköping University, Linkoping, Sweden; 4 Science for Life Laboratory, Department of Health, Medicine and Caring Sciences, Linköping University, Linkoping, Sweden; 5 Center for Medical Image Science and Visualisation, Linköping University, Linkoping, Sweden; 6 Department of Health, Medicine and Caring Sciences, Linköping University, Linkoping, Sweden

**Keywords:** Aortic Aneurysm, Echocardiography, Cohort Studies, Risk Factors

## Abstract

**Background:**

Thoracic aortic aneurysm is a life-threatening disease due to the risk for acute aortic syndromes, and subjects with dilated ascending aortas are recommended surveillance imaging to assess the need for preventive surgery. Our objectives were to investigate the progression of dilated ascending aortas and risk factors for rapid progression in a prospectively enrolled general population-based cohort of subjects aged 50–65 years.

**Methods:**

From the 5058 subjects prospectively enrolled in the general population-based Swedish CArdioPulmonary bioImage Study (SCAPIS) in Linköping, we followed all 74 subjects (22% female, mean age 59±4 years) with ascending aortic dilation (≥40 mm) identified by CT angiography, thoracic CT or transthoracic echocardiography. Office and home blood pressure (BP), pulse wave velocity, coronary artery calcification and carotid plaques were assessed at baseline. Transthoracic echocardiography was used to follow ascending aortic diameters over time.

**Results:**

Three subjects underwent acute or elective aortic repair before the first follow-up examination. Among the remaining subjects, the mean progression rate of ascending aortic diameter was 0.4 mm/year (range 0–1.8 mm/year) during a mean follow-up of 6.1±1.3 years. In 10 (14%) subjects, all men, no progression was seen. In multivariable analysis, higher 7-day home systolic BP was the only factor associated with faster progression rate.

**Conclusions:**

Progression of mild to moderate ascending aortic dilation was in general slow. Our findings emphasise the benefit of home BP measurements over office BP and underline the importance of BP control in subjects with a dilated ascending aorta.

**WHAT IS ALREADY KNOWN ON THIS TOPIC**

- Although there are prospective studies on selected cohorts of subjects with ascending aortic dilation, there are sparse data on progression rate and risk factors for rapid progression of ascending aortic dilation from a general population sample.

**WHAT THIS STUDY ADDS**

- The study shows that there is substantial variation in progression rate of ascending aortic dilation in the general population, but that progression rate is generally slow with a significant proportion of subjects who do not progress. A higher 7-day home systolic blood pressure, but not office blood pressure, was the only independent predictor of more rapid progression rate.

**HOW THIS STUDY MIGHT AFFECT RESEARCH, PRACTICE OR POLICY**

- The findings strengthen general recommendations on home blood pressure measurements and specific recommendations of blood pressure control in ascending aortic dilation. Subjects with aortic dilation and poor blood pressure control may warrant more frequent monitoring, whereas less frequent control intervals seem reasonable for subjects with continuously slow progression or stationary aortic diameters.

## Introduction

 Thoracic aortic dilation is a life-threatening condition due to the risk of aortic dissection and rupture.^1^,^3^ The ascending aorta has generally been considered dilated if ≥40 mm.^4^,^6^ In the recently published European guidelines from 2024, a lower cut-off was however suggested for ascending aortic dilatation in women.^5^ American guidelines additionally specify that an ascending aortic diameter ≥45 mm should be considered an aneurysm.^6^ Prophylactic surgery is, in patients without a genetic disorder predisposing for a higher complication risk or another concomitant disease that warrants cardiothoracic surgery, generally recommended at an aortic diameter ≥55 mm. Surgery may nevertheless be indicated at a smaller diameter in the presence of high-risk features or low surgical risk.^5 6^

Aneurysms often grow asymptomatically until complications occur. Nevertheless, aneurysms are nowadays detected at higher frequency and earlier in the course of disease, as a result of increased use of thoracic imaging and family screening. Structured, well-balanced follow-up programmes are hence required. Until recently, the European guidelines^4^ did not present any general recommendation for a follow-up strategy for sporadic, non-hereditary dilation of the ascending aorta. The recently published guidelines from 2024, however, suggest that follow-up imaging should be performed every 6–36 months depending on the degree of aortic dilation. The American guidelines^6^ from 2022 recommend surveillance imaging every 6–24 months.

An efficient follow-up strategy is indeed needed, particularly in individuals with mild to moderate ascending aorta dilation, not least to prevent excessive healthcare expenditures and to reduce radiation exposure. It seems that less dilated ascending aortas have generally slow progression rate,^3 7^ but these findings are mostly based on retrospective studies in selected cohorts. Thus, there is a lack of well-designed, prospective and general population-based studies that investigate progression of ascending aorta dilation and risk factors for dilation progression. In the ASCending Aorta study (ASCA), we prospectively follow subjects with dilated ascending aortas identified within the general population-based Swedish CArdioPulmonary bioImage study (SCAPIS).

The aim of this specific study was to describe the natural history of sporadically discovered, mildly to moderately dilated ascending aortas and to determine risk factors for progression of aortic dilation.

## Methods

### Study inclusion

SCAPIS is a nationwide study of approximately 30 000 men and women, 50–65 years old randomly selected from the general population at six sites in Gothenburg, Linköping, Malmö, Stockholm, Umeå and Uppsala (scapis.org). The principal aim of the study is to improve risk stratification for cardiovascular and chronic pulmonary obstructive disease. Study inclusion was ongoing from 2015 to 2018. The ASCA study was initiated as a local substudy within SCAPIS in Linköping.

Study subjects in SCAPIS (n=5053 in Linköping) underwent non-contrast enhanced, non-electrocardiography-gated CT of the thorax, coronary CT angiography as well as transthoracic echocardiography. According to guidelines, subjects with ascending aortic diameter ≥40 mm at any of the three examinations were considered for inclusion in the present study.^4^,^68^ Maximum ascending aortic diameter in these subjects was re-evaluated based on multiplanar reformats of the non-contrast, non-electrocardiography-gated CT chest images at 1 mm slice thickness on an IDS7 workstation.

Additional recordings from the SCAPIS inclusion comprise coronary CT for calcium scoring, office blood pressure (BP) and home BP, pulse wave velocity and carotid ultrasound, as described below.

### Baseline and follow-up echocardiography in ASCA

**Figure 1:**
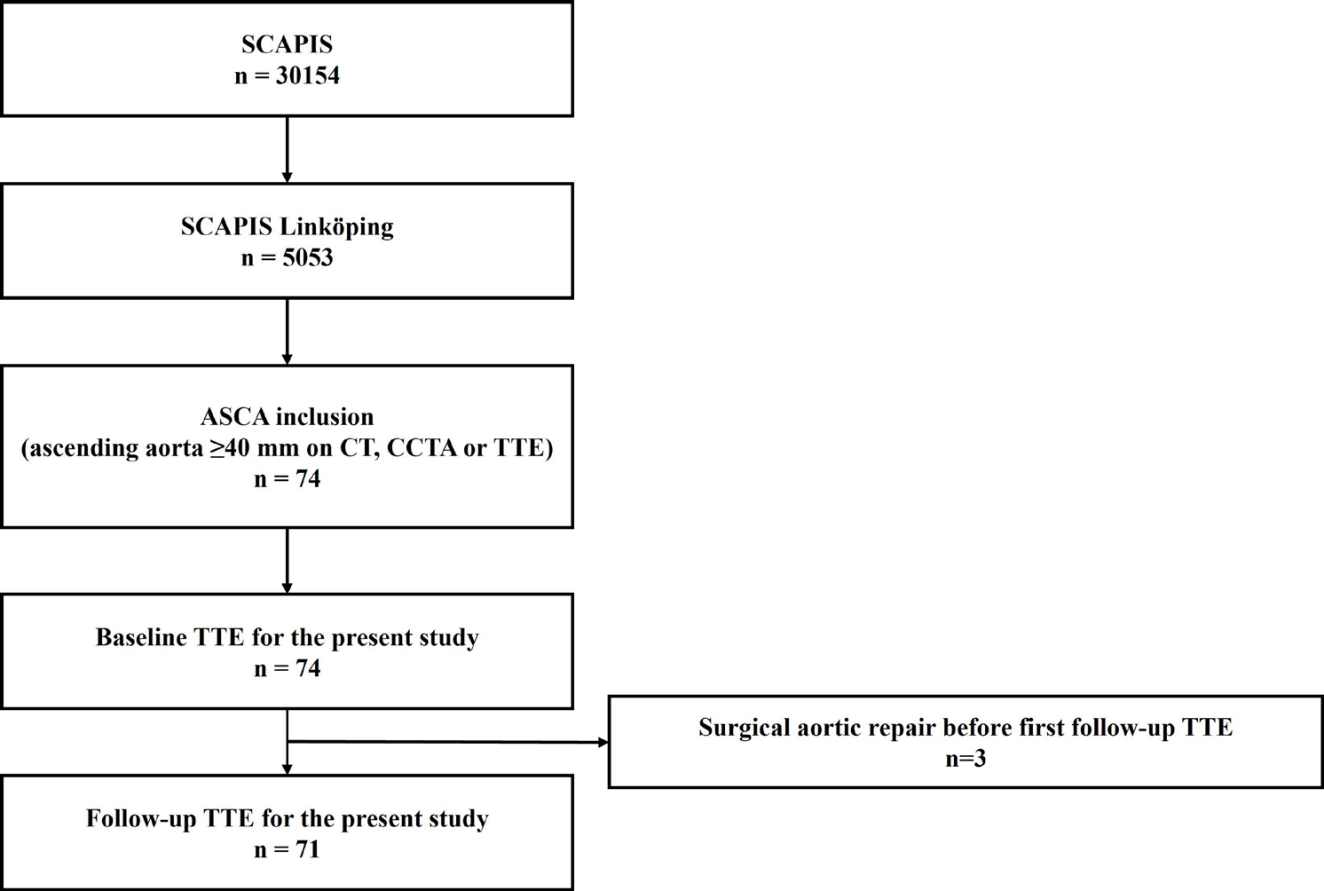
Flow chart illustrating study inclusion as well as baseline and follow-up examinations for the present study. ASCA, ASCending Aorta study; CCTA, coronary CT angiography; SCAPIS, Swedish CArdioPulmonary bioImage Study; TTE, transthoracic echocardiography.

After inclusion, all subjects underwent an additional echocardiographic evaluation with particular focus on the ascending aorta. This examination was used for baseline aortic diameter measurements in this study. The baseline echocardiography was performed 11±10 months after the SCAPIS core examination. Subsequent follow-up echocardiographic examinations were thereafter performed regularly for evaluation of aortic dilation progression. Reported baseline and follow-up aortic measurements are hence from the same imaging modality, that is, transthoracic echocardiography (TTE). Echocardiographic examinations were performed at the Department of Clinical Physiology at Linköping University Hospital, Sweden, using Vivid E95 Echocardiography Systems, initially with M5Sc and later 4VC probes (GE Healthcare, Horten, Norway). Study inclusion, baseline and follow-up examinations for the present study are depicted in figure 1.

Measurements of the aortic root at the level of the sinus of Valsalva and the tubular ascending aorta were performed in the parasternal long-axis view by measuring the largest leading edge-to-leading edge diameter, according to recommendations.^9^ Aortic valve morphology was assessed in short-axis views at the level of the aortic valve. Aortic stenosis and aortic regurgitation were graded according to recommendations.^10 11^ One experienced examiner performed all measurements and evaluated aortic valve morphology and function. Aortic growth rate was calculated as the difference between the diameter at the first examination and the diameter at the latest examination divided by follow-up time in years. For subjects presenting with a negative change, the value was considered as 0 (no growth).

### Coronary CT for calcium scoring

A dedicated CT scanner (Somatom Definition Flash scanner, Stellar detector, Siemens Healthineers, Forchheim, Germany) was used to acquire spiral images of the chest. Image acquisition in SCAPIS has previously been described.^12^ Coronary artery calcium content was determined for each coronary artery in all but one subject using electrocardiogram-gated, non-contrast CT imaging and total Coronary Artery Calcium Score (CACS) was calculated according to recommendations.^12^

### BP and pulse wave velocity

Office BP was measured twice by an automatic device (Omron M10-IT, Omron Health Care, Co., Kyoto, Japan). Mean systolic and mean diastolic BP were recorded. The same automatic device was used to measure home BP twice a day for 1 week, as previously described.^13^ In short, subjects were asked to measure BP in the seated position in the morning and evening for 7 days except for the first day when no morning measurements were done. In total, 39 home BP recordings (each session was based on the mean of three measurements with 1 min apart) were made by the subjects at these 13 occasions.

Carotid-to-femoral pulse wave velocity was measured as previously described^14^ using a cuff-based SphygmoCor Xcel device (Atcor Medical, Australia) in 51 (68%) of the subjects. The average of two measures was used.

### Carotid ultrasound

Carotid arteries were assessed for atherosclerotic plaques in the common carotid, bulb and internal carotid artery using a standardised protocol and a Siemens Acuson S2000 ultrasound scanner with a 9L4 transducer (Siemens Healthineers, Forchheim, Germany). Plaques were, according to recommendations,^15^ defined as focal structures intruding into the arterial lumen of ≥0.5 mm height or 50% of surrounding intima–media thickness, or as a thickness of >1.5 mm measured from the intima–lumen interface to the media–adventitia interface. Significant carotid atherosclerosis was defined as >1 plaque, as previously described.^16^

### Statistics

Statistical analyses were performed using SPSS V.27 (IBM, Armonk, New York, USA). Subjects were grouped as having a fast progression rate if it was above the 90th percentile. The Student’s t-test or one-way analysis of variance were used for group comparison of continuous variables. For group comparisons of categorical variables, Fischer’s exact test was used. After testing for collinearity, we included the following variables in a multivariable linear regression model to investigate their association with progression rate (fastest progression rate at either root or tubular ascending aorta): age, sex, current smoker, heredity of aortic aneurysm, dissection or rupture, largest baseline ascending aorta diameter, bicuspid aortic valve, office systolic BP, mean home systolic BP, CACS>0 and carotid plaques. Continuous variables are expressed as mean±SD or range and categorical values as number (per cent). A p value<0.05 was considered statistically significant.

### Patient and public involvement statement

Patients and/or the public were not involved in the design, or conduct, or reporting or dissemination plans of this research.

## Results

Seventy-four subjects with ascending aorta dilation were included in the study, 16 (22%) were female and mean age was 59±4 years. Baseline aortic root and tubular ascending aorta diameters were 40±4 mm (range 32–50 mm) and 43±3 mm (range 32–54 mm), respectively. Forty (54%) subjects had concomitant dilation of the aortic root and tubular ascending aorta. In 18 (24%) cases, the aortic root was larger than the tubular ascending aorta. Bicuspid aortic valve was present in nine (12%) subjects.

Three subjects underwent surgical aortic repair (two prophylactic surgery and one acute surgery) within 1–1.5 years from the baseline examination and were not included in the follow-up. All other subjects were followed with a mean follow-up time of 6.1±1.3 years. Three subjects underwent surgical aortic repair during follow-up. One subject could not be followed by TTE due to poor image quality, and another was lost to follow-up after 3 years.

### Progression of aortic dilation

The greatest mean yearly progression at either root or tubular ascending aorta was 0.4 mm (range 0–1.8 mm), with 10 (14%) subjects, all men, who did not progress. The mean yearly progression rate of the tubular ascending aorta was 0.4 mm (range 0–1.8 mm), with 12 (17%) subjects who did not progress. The mean yearly progression rate of the aortic root was 0.2 (range 0–0.9 mm), 28 (40%) subjects did not progress. Progression rates did not differ between sexes (0.4 mm/year for both sexes, p=0.762).

Among subjects with greater diameter in the tubular ascending aorta than in the aortic root (n=50), a majority (64%) had the fastest growth rate in the tubular ascending aorta. Only 12% had a faster growth rate in the root, whereas there was no difference in growth rate between root and tubular ascending aorta in 14%, and 10% did not grow. Among subjects with greater diameter in the aortic root than in the tubular ascending aorta (n=17), 24% had the fastest growth rate in the root, whereas 41% still had a faster growth rate in the tubular ascending aorta. For 12% there was no difference in growth rate between root and tubular ascending aorta, and 24% did not grow.

### Characteristics of subjects with faster, slower or no growth and risk factors for rapid progression

Characteristics of subjects with faster, slower or no growth are presented in table 1. Subjects with fast growth had higher 7-day mean systolic BP in the morning and evening. At least moderate aortic regurgitation was more common among subjects with fast progression. In multivariable linear regression, 7-day home systolic BP was the only variable with significant association to progression rate (table 2).

**Table 1:** Characteristics of subjects with no, slow or fast aortic growth

	No growth (n=10)	Slow growth (n=53)	Fast growth* (n=7)	P value
Age, years	61±4	59±4	62±2	**0.033**
Female sex, n (%)	0 (0%)	14 (26%)	1 (14%)	0.141
Current smoker, n (%)	2 (20%)	4 (8%)	1 (14%)	0.271
Heredity for aortic aneurysm, dissection or rupture, n (%)	2 (20%)	6 (11%)	1 (14%)	0.294
Aorta and aortic valve characteristics				
Baseline tubular ascending aorta diameter, mm	42±4	44±3	42±2	0.221
Baseline tubular ascending aorta diameter index, mm/m^2^	20±2	21±3	21±3	0.493
Baseline aortic root diameter, mm	41±4	40±4	40±6	0.907
Baseline aortic root diameter index, mm/m^2^	19±2	19±2	20±4	0.727
Largest aortic root or tubular ascending aorta diameter ≥45 mm, n (%)	5 (50%)	24 (45%)	2 (29%)	0.779
Bicuspid aortic valve, n (%)	0 (0%)	6 (11%)	0 (0%)	0.786
Aortic valve prosthesis, n (%)	0 (0%)	1 (2%)	0 (0%)	1.000
At least moderate aortic regurgitation, n (%)	0 (0%)	0 (0%)	2 (29%)	**0.009**
Aortic valve calcification, n (%)	3 (30%)	7 (13%)	0 (0%)	0.150
Aortic stenosis, n (%)	0 (0%)	2 (4%)	1 (14%)	1.000
Blood pressure and pulse wave analysis				
Hypertension, physician-diagnosed, n (%)	5 (50%)	16 (30%)	4 (57%)	0.214
Antihypertensive drug treatment, last 2 weeks, n (%)	4 (40%)	14 (26%)	3 (43%)	0.826
Systolic blood pressure, mm Hg	138±19	137±14	141±19	0.864
Diastolic blood pressure, mm Hg	86±9	87±8	87±14	0.943
Pulse pressure, mm Hg	51±10	50±9	54±11	0.576
7-Day home mean systolic blood pressure, mm Hg				
Morning	132±14	123±11	139±13	**0.002**
Evening	128±15	125±11	139±17	**0.025**
7-Day home mean diastolic blood pressure, mm Hg				
Morning	84±9	83±8	85±6	0.678
Evening	81±6	82±7	85±12	0.658
Pulse wave velocity, m/s	10.3±2.2	9.2±1.5	9.9±1.3	0.216
Pulse wave velocity >10 m/s, n (%)	3 (30%)	12 (23%)	2 (29%)	0.477
Atherosclerosis burden				
Coronary artery calcium score >0, n (%)	3 (30%)	32 (60%)	5 (71%)	0.287
Carotid plaques, n (%)	8 (80%)	25 (47%)	5 (71%)	**0.032**

Statistically significant results are in bold

* Subjects were grouped as having a fast progression rate if it was above the 90th percentile.

**Table 2:** Associations between aortic dilation progression rate and potential risk factors in a multivariable linear regression model

	B (95% CI)
Age	0.002 (−0.017 to 0.021)
Female sex	−0.053 (−0.259 to 0.153)
Current smoker	−0.114 (−0.441 to 0.214)
Heredity for aortic aneurysm, dissection or rupture	−0.003 (−0.232 to 0.226)
Baseline aortic root or tubular ascending aorta diameter	−0.029 (−0.061 to 0.002)
Bicuspid aortic valve	−0.040 (−0.358 to 0.279)
Office systolic blood pressure	−0.005 (−0.014 to 0.003)
7-Day home mean systolic blood pressure	**0.012 (0.001 to 0.023**)
Coronary artery calcium score >0	0.061 (−0.096 to 0.217)
Carotid plaques	0.010 (−0.151 to 0.171)

Statistically significant data is presented in bold.

## Discussion

The finding in our prospective, general population-based study of a mean progression rate of only 0.4 mm/year in mildly to moderately dilated ascending aortas is in line with other modern-day, prospective^17^,^19^ and retrospective^20^,^22^ studies in selected cohorts of subjects with dilated ascending aortas. The prospective, general population-based Danish Cardiovascular Screening Trials (DANCAVAS), nevertheless, includes a comprehensive evaluation of approximately 650 subjects with aortic dilation from the general population and reports both progression rate of ascending aorta diameters and the incidence of acute aortic events.^3^ The progression rate of aortic diameters in DANCAVAS was approximately 0.1 mm/year and although greater diameters were associated with increased risk for aortic events, they surprisingly did not find that large aneurysms progressed faster. The DANCAVAS population was larger than ours, approximately 10 years older, included only subjects with tricuspid aortic valves and heredity for aortic disease was reported less frequently than in our population. While we used the clinical standard methodology with TTE for follow-up of aortic diameters, electrocardiogram-gated non-contrast CT was used in DANCAVAS.

In our study, the mean progression rate was approximately four times that of a normal aorta,^23^ but there was substantial variability with a significant proportion of individuals who did not progress at all and others who had a more rapid progression rate. The greatest progression rate noted in our study was 1.8 mm/year, which is in line with others^3 17^ who also found that a yearly progression rate of >2 mm was extremely rare in 40–50 mm wide ascending aortas. Wu *et al*^24^ found the mean yearly progression rate of dilated ascending aortas to be greater, 1 mm/year, when analysing a large, but retrospectively collected material from a dedicated aorta clinic. However, the largest yearly progression rate was no greater than 1.8 mm/year in their cohort either. The recently published European guidelines^5^ recommend that follow-up intervals for sporadically discovered ascending aortic dilation should be based on aortic diameter. It is suggested that mild dilation of up to 44 mm should be re-evaluated by TTE every 2–3 years and that ascending aortic dilation of 45–49 mm should be re-evaluated yearly if progression rate is not very rapid (≥3 mm/year), when follow-up is recommended every 6 months. American guidelines^6^ recommend surveillance imaging every 6–24 months. Our findings underscore the reasonableness of less frequent follow-up intervals in mild ascending aortic dilation and indicate that an even sparser follow-up may be rational in subjects with stable diameters or continuously slow progression rate. Larger aneurysms have been found to grow faster,^24^ and more frequent monitoring is warranted to effectively time preventive surgery. Interestingly, the progression rate was most often, but not always, greatest at the site of the largest dilation, which underlines the importance of considering the entire length of the ascending aorta during follow-up.

Importantly, even though office BP was not higher in subjects with rapid progression of the aortic dilation, they had higher, and on average elevated, systolic BP on 7-day home BP measurements. In fact, using multivariable linear regression, a higher systolic BP on 7-day home BP measurements was the only factor associated with progression rate. Although BP has been linked to thoracic aortic dilation,^8^ neither office BP nor a diagnosis of hypertension has been associated with progression rate.^19 22^ Home BP measurements appear to be better associated with cardiovascular risk than office BP in various populations^25^ and recent guidelines recommend a wider use of home BP measurements.^26^ Our findings indicate that home BP measurements may be of specific value in monitoring BP in subjects with a dilated ascending aorta and emphasise present recommendations of BP control.^5 6^ More frequent monitoring may be appropriate in subjects with poor BP control.

Subjects with rapid progression more often had at least moderate aortic regurgitation, which is coherent with another study.^20^ More than mild aortic regurgitation was, nevertheless, very rare and only present in two subjects. Previous studies on the association between atherosclerosis and thoracic aortic dilations are diverging,^27 28^ and to our knowledge, no previous study has investigated the relationship between atherosclerosis and progression of aortic dilation. Whereas the frequency of carotid plaques differed between individuals with no, slow or rapid progression in our study, there was no association between atherosclerosis and growth rate in the multivariable linear regression.

Notably, although the progression rate of aortic dilation did not differ between sexes, all subjects in whom the dilation did not progress were men. There are previous studies which indicate more rapid progression of ascending aorta dilation in women^3 21 22 29^ and female sex has been associated with a higher risk for acute aortic syndromes.^30^ Women are however generally smaller than men and aortic dimensions are related to body size.^23^ It is plausible that an aortic diameter ≥40 mm in women may more often indicate aortic disease, as is also suggested in the new European guidelines where the upper limit of normal for the aortic root and tubular ascending aorta in women are set as low as 34 and 36 mm, respectively.^5^ Sex differences in thoracic aortic disease are nevertheless poorly understood, and unknown factors may contribute to the observations that do not seem to be fully accountable to differences in body size.^29^

### Strengths and limitations

The present study is a prospective, general population-based study of dilated ascending aortas and includes an extensive characterisation of the population. The mean follow-up time of approximately 6 years is longer when compared with other prospective studies in selected populations. SCAPIS was, however, not designed to primarily evaluate aortic diameters and cases were identified based on aortic diameters measured on non-contrast, non-electrocardiography-gated CT thorax, coronary CT angiography or TTE. Nevertheless, to harmonise baseline measurements in-between participants and measurements from baseline to follow-up within a participant, all subjects included in the study subsequently underwent TTE and baseline as well as follow-up aortic diameters in the present study are from the TTE examinations. Although based on screening of aortic diameter in around 5000 individuals, our study is limited by the small study population, not least since power may have been too low to identify all associations between potential risk factors and progression rate. Another limitation is the narrow age span of 50–65 years, which, however, represents an age span in which many individuals undergo cardiovascular imaging and thus a time-point when dilated ascending aortas may frequently be detected. Study inclusion was based on an absolute aortic diameter of ≥40 mm. This was deemed to be reasonable from a clinical perspective, but we acknowledge that aortic dimensions indeed vary with age, sex and body size and optimal, individually adapted cut-off values for a dilated aorta may still need to be determined. Importantly, although some subjects had a family history of aortic disease, no subject had a known genetic disease predisposing for thoracic aortic disease other than bicuspid aortic valves. Our findings hence do not apply to patients with such genetic diseases and we underline that in subjects with family history of aortic disease, screening for hereditary disorders and syndromes should be considered. Finally, as stated above, subjects included in the study had generally mild to moderate ascending aortic dilation and larger aortic aneurysms may indeed grow faster.

## Conclusions

In this general population-based prospective study of subjects 50–65 years, we conclude that the mean yearly progression rate of mildly to moderately dilated ascending aortas is generally slow and a significant number of subjects do not progress at all. There are nevertheless substantial variation in progression rate and a higher 7-day home systolic BP was the only clinical parameter independently associated with more rapid progression of the dilation. This emphasises the use of home BP measurements and current recommendations of BP control in subjects with ascending aortic dilation. Subjects with poor BP control may warrant more frequent monitoring, whereas less frequent control intervals seem reasonable for subjects with continuously slow progression rate or even stationary aortic diameters.

## Data Availability

Data are available upon reasonable request. Data may be obtained from a third party and are not publicly available.
